# A Rare Case of Cocaine-Induced Muscle Hematoma Followed by Rhabdomyolysis in Association With Acute Kidney Injury and Severe Transaminitis

**DOI:** 10.7759/cureus.25035

**Published:** 2022-05-16

**Authors:** Arjun Mainali, Tutul Chowdhury, Samaj Adhikari, Navodita Uprety, Pharlin Noel, Nicole Gousy

**Affiliations:** 1 Internal Medicine, Interfaith Medical Center, New York, USA; 2 Surgery, Mount Sinai South Nassau Hospital, Oceanside, USA; 3 Medical School, American University of Antigua, New York, USA

**Keywords:** transaminitis, drug induced acute kidney injury, cocaine use, a complication, muscle hematoma

## Abstract

Cocaine use can result in a few infrequent complications, among which is localized hematoma. As far as we know, there are very few cases reporting cocaine-induced non-traumatic muscle hematoma complicated by severe transaminitis and rhabdomyolysis. Here we present a patient who developed a significant lower extremity muscle hematoma secondary to inhalational cocaine use. We are reporting this case to bring to light this rare and unusual complication of cocaine use. With recreational use of this drug being so prominent in the patient population, early recognition of this complication can help expedite treatment and reduce the severity of end-organ damage.

## Introduction

Cocaine use is a huge public health burden in the USA [1]. There is a wide spectrum of medical complications associated with cocaine use, notably myocardial ischemia, cerebral ischemia, and aortic dissection, to name a few [1]. It is well known that cocaine-induced arterial vasoconstriction is the cause of these aforementioned complications. 

A significant proportion of cocaine users experience muscle injuries, which are attributed to cocaine-induced vasospasms propagating muscle ischemia and infarction [1,2]. Intracerebral hemorrhage and alveolar hemorrhage secondary to cocaine use are also common complications seen after frequent cocaine use [1,3-5]. However, cocaine-induced muscle hematoma is rarely seen and has been scantily reported in the literature [6]. We present a case of a young female who presented with lower extremity pain after cocaine inhalation, with subsequent MRIs revealing muscle hematoma in addition to rhabdomyolysis with secondary renal failure requiring hemodialysis.

## Case presentation

A 33-year-old female with a past medical history of alcohol use disorder, polysubstance abuse, and major depressive disorder was brought in by emergency medical services because of pain and weakness in her left thigh for one day. She noticed it after waking up in the morning, and since waking up, it gradually worsened throughout the day. She denied any recent trauma, numbness, tingling, weakness in any other body part, fall, insect bite, or any appreciable swelling or redness. She also denied back pain and bowel or bladder incontinence. Other reviews of systems were unremarkable. She has a ten-year smoking history with a pack-year of five and a daily alcohol intake of 1-2 pints of vodka for more than one year. She also admitted to smoking cocaine "on and off", with the last cocaine intake occurring the night before the symptoms started. 

On examination: she was in mild distress due to pain. Triage vitals included: blood pressure of 92/68, heart rate of 85, temperature 36.7 °C (98 °F), respiratory rate 17, with an oxygen saturation of 99% on room air. Physical exam was significant for tenderness on the left hip with a restricted range of motion due to pain. No findings of trauma like skin bruises and ecchymosis were noted and appropriate lab work was performed (Tables 1-4).

**Table 1:** Results of the patient’s complete blood count (CBC), complete metabolic panel (CMP), coagulation profile, toxicology profile, hepatitis serology panel and autoimmune panel. WBC: white blood cell count  Hg: hemoglobin; BUN: blood urea nitrogen; AST: aspartate aminotransferase; ALT: alanine aminotransferase; ALP: alkaline phosphatase; CO2: carbon dioxide; CK: creatine kinase, LDH: lactate dehydrogenase, CRP: C-reactive protein, PT: prothrombin time, INR: international normalized ratio; PTT: partial thromboplastin time, Ab: antibody, HEV: hepatitis E virus, HbsAG: hepatitis B surface antigen, HEP BE AG: hepatitis B E antigen, ANA: antinuclear antibody

Test	Ref Range and Units	Values
WBC	4.5-11.0 10x3/uL	15.7
Hb	11.0-15.0 g/dL	12.4
Platelets	130-400 10x3/uL	361
BUN	7.0-18.7 mg/dL	24
Creatinine	0.57-1.11 mg/dL	2.42
Na	136-145 mmol/L	139
K	3.5-5.1 mmol/L	4.9
CO2	22-29 mmol/L	23
Total Bilirubin	0.2-1.2 mg/dL	0.7
ALT	10-55 U/L	3195
AST	5-34 U/L	>3600
ALP	40-150 U/L	88.7
Albumin	3.5-5.2 g/dL	4.4
CK	29.0-168.0 U/L	>36000
LDH	125-220 U/L	>3600
Ca	8.4-10.2 mg/dL	7.5
Phosphorus	2.3-4.7 mg/dL	6.5
Uric acid	2.5-6.2 mg/dL	6.7
Lactate	0.50-1.90 mmol/L	2.95
CRP	0.50-1.00 mg/dL	3.7
PT	9.8-13.4 sec	14.4
INR	0.85-1.15	1.18
PTT	24.9-35.9	28.2
Ethanol	0.00-14.00 mg/dL	4.2
Salicylate level	15.0-29.9	<10
Acetaminophen, serum	10.0-30.0 ug/ml	<0.7
Ammonia	18.0-72 umol/L	61.7
Hepatitis A total Ab	Negative	Equivocal
HEV IgG	Negative	Negative
HbsAG	Negative	Negative
Hepatitis B core total AB	Negative	Negative
Hepatitis B Core IgM	Negative	Negative
HEP BE AG	Negative	Negative
Hepatitis C	Negative	Negative
ANA	Negative	Negative
Actin (smooth Muscle) Ab	0-19 units	8
Mitochondrial (M2) Ab	0.0-20.0 units	<20

**Table 2:** Patient’s urinalysis taken during her admission WBC, white blood cells; RBC, red blood cells; HPF, high power field

Test	Ref Range and Units	Values
Color	Light yellow	Red
Clarity	clear	clear
Specific gravity	1.005-1.030	>1.030
pH	5.0-8.0	6.5
Glucose	0 mg/dL	Negative
Protein	0 mg/dL	>300
Blood	Negative	Large
Nitrite	Negative	Negative
Leucocyte esterase	Negative	Negative
WBC	0-5/HPF	0-5
RBC	0-4/HPF	4-10

**Table 3:** Patient's toxicology screening taken during her admission

Test	Ref Range and Units	Values
Urine cocaine	Negative (cutoff <300 ng/ml)	Positive
Cannabinoid	Negative	Negative
Barbiturate	Negative	Negative
Benzodiazepine	Negative	Negative
Methadone	Negative	Negative
Propoxyphene	Negative	Negative
Ethanol	Negative	Negative
Opiate	Negative	Negative
Phencyclidine	Negative	Negative

**Table 4:** Patient's viral serology panel taken during her admission

Test	Ref Range and Units	Values
SARS-Cov-2 PCR	Not detected	Not detected
Influenza A, NAA	Not detected	Not detected
Influenza B, NAA	Not detected	Not detected

**Figure 1:**
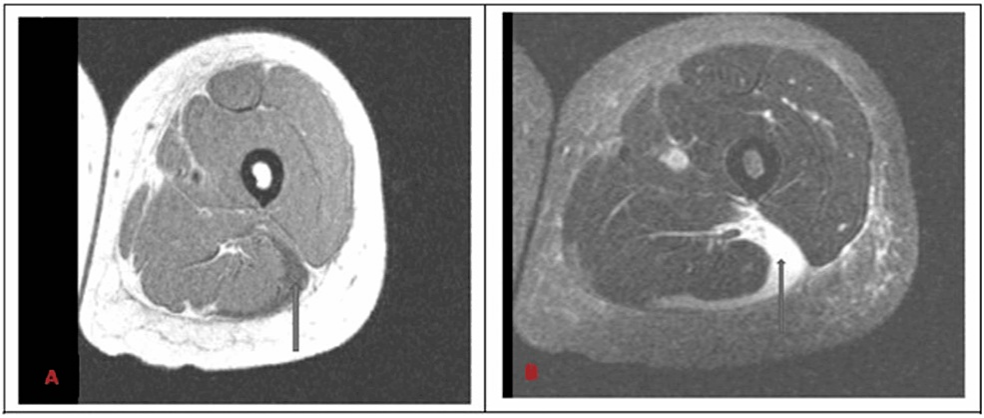
Axial T1 (A) and T2(B)-weighted MRI of left lower extremity showing large hematoma/fluid tracking in the fascial planes surrounding lateral biceps femoris muscle. The blue arrows are pointing to the hematoma

**Figure 2:**
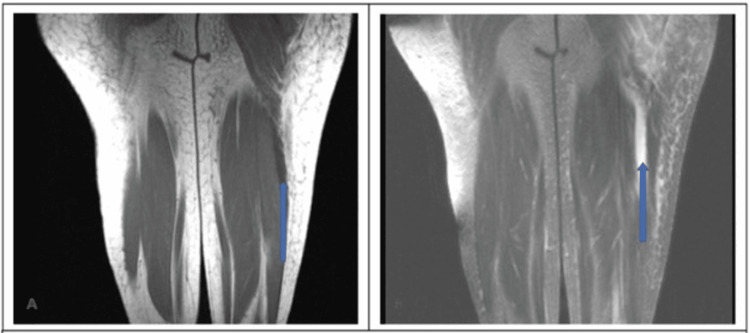
Coronal T1 (A) and T2 (B)- weighted MRI of the left lower extremity showing a large hematoma/fluid tracking in the fascial planes surrounding lateral biceps femoris muscle (blue arrows)

An X-ray of the left hip did not show any fractures or dislocations and the ultrasound of the abdomen was unremarkable. There was no change in limb girth, skin tension, or any signs suggestive of elevated compartmental pressure; however, she had persistent pain and tenderness on the left hip with restricted range of motion which prompted a CT scan of the lower extremity. The CT scan was inconclusive and could not explain the physical finding of the patient, so an MRI was done. A CT of the left lower extremity was suspicious for lower hairline nondisplaced fracture of the greater trochanter but repeat CT and MRI of the left lower extremity were negative for fracture. An MRI of the left lower extremity showed a large hematoma with fluid tracking in the fascial planes (Figures 1-2). CT of the cervical, thoracic and lumbar spine were unremarkable.

**Figure 3:**
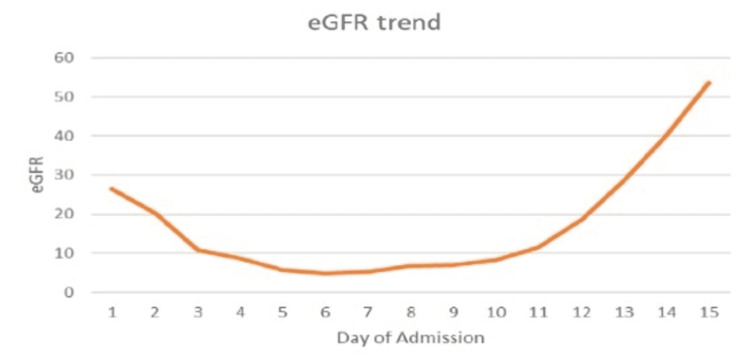
eGFR trend of the patient during the course of her hospital stay eGFR: estimated Glomerular Filtration Rate

The patient was initially managed with pain medication for muscle hematoma and was started on the N-acetylcysteine infusion protocol for severe transaminitis associated with rhabdomyolysis. She also received boluses of fluid with continuous IV hydration and was started on a sodium bicarbonate drip to alkalinize the urine at the rate adjusted to achieve a urine pH of >6.5. Her blood pressure improved with hydration. Strict intake/output charting was done. Despite these measures, her acute kidney injury (AKI) kept worsening (Figure 3: eGFR trend of the patient during the hospital stay), and she underwent one session of dialysis on the sixth day of admission. In contrast, her serum aminotransferases level and creatinine kinase (CK) were trending downward. Her kidney function started improving without further dialysis, and serum aminotransferases level and CK also trended down to normal. During her diuretic phase of AKI she had hypokalemia which was supplemented accordingly. She was offered hematoma evacuation surgery after her lab functions improved, but she refused the surgery and chose to go with conservative management.

## Discussion

Cocaine is a highly addictive stimulant substance derived from the leaves of the Erythroxylum coca plant, which grows predominantly in Peru, Bolivia, and Ecuador [1,7]. Cocaine inhibits catecholamine reuptake in presynaptic neurons in both the central and peripheral nervous systems, increasing catecholamines, sympathetic output, and stimulation [8]. Cocaine increases the dopamine level in the brain in the mesolimbic pathway, also known as the "reward pathway," and the nigrostriatal pathway by hindering dopamine recycling. This causes enormous amounts of this catecholamine to accumulate between the nerve cells, preventing normal transmission [1]. This dopamine rush in the brain's reward system then reinforces drug use. While the reward circuit may adapt over time, the euphoric effects of the drug will dampen as the dopaminergic neurons become less sensitive to the medication; this subsequently causes a progressively weaker dopaminergic surge to occur. As a result, users take higher and more frequent doses to achieve the same high and avoid withdrawal [1].

Cocaine, in comparison to other illicit drugs, is one of the most commonly used illicit drugs, with the greatest rates among men aged 35-44 years, resulting in a massive social and economic burden [8]. Cocaine abuse has been associated with intracerebral and subarachnoid hemorrhage, spontaneous acute subdural hematoma, cerebrovascular abnormalities, with cardiovascular complications as the most documented [8]. Cocaine has additionally been linked to stroke, particularly in younger people between 15 and 44 years old [7]. Cocaine-induced splenic hematoma is also a serious complication that has been documented [9]. Splenic hemorrhage can occur as a result of arteriolar rupture caused by rapid hypertension or as a result of infarction and subsequent hemorrhage following vasospasm resolution [9]. Our patient, however, presented with a spontaneous large hematoma in the fascial planes surrounding the lateral left biceps femoris muscle one day after using cocaine which, due to the rarity of the complication, the pathogenesis resulting in this presentation remains unknown.

It is well documented that cocaine causes harm to the cardiovascular and cerebrovascular systems and repeated use can lead to significantly increased risks for the development of vascular disease. The exact mechanism of action of how cocaine increases vascular disease has been well studied. Cocaine's acute and chronic vascular effects are multifaceted, involving hypertension, altered homeostasis and platelet function, thrombosis, thromboembolism, and changes in blood flow [8]. Cocaine's acute hematological effects on the vascular system result in the loss of protective endothelium activities [8]. This is due to the drug’s ability to induce the release of endothelin-1, a vasoconstrictor protein generated by vascular endothelial cells when vessels are stressed, and a decrease in nitric oxide (a blood vessel dilator) resulting in vasoconstriction [8]. Furthermore, the effects of cocaine on vasoconstriction are associated with increased calcium levels in the vessels [7]. Cocaine's acute stress on the endothelium of the vessel may result in aneurysm formation. Additionally, the potent vasoconstrictive impact of cocaine may result in an abrupt elevation in vascular pressure. Individuals who have an underlying arteriovenous malformation or aneurysm are more likely to experience such episodes. We believe when these two factors combine, the chance of vascular rupture increases, which can result in hematoma development in any organ. 

Cocaine consumption has a number of known pathophysiological effects that may also increase the risk of aortic dissection [10]. The aorta's elastic characteristics have been shown to change with chronic cocaine consumption, resulting in a decrease in aortic strain and distensibility along with an increase in the aortic stiffness index [10]. Our patient had a long history of cocaine abuse and was prone to chronic changes similar to those seen in aortic dissection. These chronic changes might also play a role in her presenting symptoms. 

Our patient also developed rhabdomyolysis and AKI, a well-known complication of cocaine use. In 1987, the first case of rhabdomyolysis linked to acute cocaine consumption was published in the medical literature [11]. Cocaine can decrease oxygen delivery to any organ due to its vasoconstrictive properties, which can result in significant end-organ damage. When muscles are damaged, myoglobin is released and filtered through the glomerulus. AKI can then be perpetuated by myoglobin heme-induced renal tubular damage in the setting of cocaine-induced rhabdomyolysis, as seen in our patient [12].

## Conclusions

In our extensive review of English medical literature, we could find only a few cases of reported muscle hematoma due to cocaine abuse. Spontaneous muscle hematoma is not a well-known complication of cocaine abuse, but this case highlights the fact that clinicians should be aware of this association, especially considering how endemic cocaine use is among the population. Early diagnosis and treatment could prevent further downstream complications like rhabdomyolysis and compartment syndrome, necessitating the importance of early identification of these complications.
